# The Hospitals of Bristol

**Published:** 1890-11-08

**Authors:** 


### THE HOSPITALS OF BRISTOL.

The situation of Bristol is such as to enable it to command the whole of the west and south-west of England. If any section of Bristol people wished to lead public opinion in the west there is no city more favourably placed for the successful enforcement of sound views and wise reforms. Of course, so unique a position carries with it proportionate temptation, and, although it has had enlightened representatives in Parliament for many years, still on the whole, as a city it is essentially conservative—slow to move and difficult to persuade when changes are not only desirable but necessary. In the past its citizens have been pioneers in medical matters, for the Bristol Royal Infirmary dates back to the year 1735, and so may be regarded as one of the earliest, if not the earliest of provincial English hospitals. No doubt the knowledge that, with the exception of the Children's Hospital, the hospital buildings belong to a period when hospital construction was in its infancy has led many people to conclude that there was little to learn and nothing novel to study in this respect in so ancient a city. Fortunately Bristol presents at the present time the pleasant spectacle of a united medical profession, zealous in good works, active in scientific research, and numbering amongst its members those who have much more than a local reputation. To this latter circumstance one may credit in a great measure the marked improvement in the hygienic condition and the administrative efficiency of the Royal Infirmary and the Bristol General Hospital. These institutions strike the expert as being conducted upon sound principles, whereby the best has been made of the existing buildings, so that the results of the medical and surgical treatment within their walls at the present time leave little to cavil at, whilst they exhibit much to commend. Of course there are defects due to original errors at the time when the first buildings were put up, but it is only fair to say, that thanks to the zeal and ability of the medical and resident staff, and to the intelligence and interest displayed by the governors in whom the management is immediately vested, both the infirmary and the hospital are well suited for the purposes to which they are at present devoted.

### The Royal Infirmary.

This institution was established in the year 1735. Originally occupying an old house, the pressure upon the accommodation speedily necessitated the erection of a hospital, which was modelled on what is known as the straight plan. From time to time additions have been made, so that the original design has now been converted into a U, with the addition of a large out-patient department, which has been placed in the centre and at the back of the original buildings. It is matter for congratulation that the authorities have erected a Nurses' Home, where the nursing staff now sleep, on a plot of ground about five minutes' walk from the hospital. They have converted the old bedrooms attached to the wards into sitting-rooms for the staff nurses, recognising that it is not only undesirable but unfair to allow a charge nurse to have her rest disturbed and her health impaired by sleeping in a room in juxtaposition to the ward over which she has charge. In this respect the old Bristol Infirmary is far and away in advance of the new Royal Infirmary at Liverpool which was opened the other day, seeing that the architect has disregarded the teaching of experience by placing the sleeping rooms of the sisters in juxtaposition to the wards. There is an excellent dining-room for the nurses in the basement of the building, and the staff now consists of about sixty specially selected, and highly-trained nurses. It is, however, matter for surprise that, owing possibly to the difficulty of providing accommodation, no proper sitting or recreation room has yet been set apart for the nursing staff. Having regard to the fact that such a provision is essential to the well-being and comfort of the nurses, we make no doubt that the Ladies' Committee will take up this matter, and then a nurses' library and sitting-room, adequately furnished and pleasantly situated, will soon be forthcoming. The wards as a whole are bright and cheerful, and when the present intention of reducing the beds in each ward to ten has been carried out, they will leave little to criticise and much to praise. Dr. Swayne, the senior medical officer, has laboured assiduously to improve the hygienic conditions, and has effected many administrative reforms in various directions. The conversion of the upper storey of the hospital into an ophthalmic department has been admirably done, and the operation theatre is especially good. There is a good-sized garden in the rear of the hospital, where the residents are enabled to get both air and exercise, a lawn-tennis court being also provided. Miss M. D. Smith, the matron, is deservedly popular, and her management has produced much that is noteworthy and satisfactory.

### Bristol General Hospital.

This institution was not erected until 1832, or about a century later than the infirmary. It is situated close to the docks, and mainly ministers to the needs of those engaged therein. The hospital is erected on the L plan, and recently a new wing consisting of wards and a nursing home has been added. Formerly the floors were of asphalte but these have nearly all been replaced by oak, whereby the sanitary condition of the hospital and the comfort and health of the patients have been greatly benefited. About four years ago the whole of the drainage system was reconstructed and the results of the treatment at the present time are very good. None of the nurses sleep in the rooms adjacent to the wards which have been converted into nurses' sitting rooms. Nearly every nurse is now provided with a separate bedroom in the new home, which will well repay a visit. The general sitting-room is tastefully fitted and contains a good library and other comforts, including a piano, all calculated to promote the nurses' happiness when off duty. The nurses dining-hall is situated in the hospital proper, and is a large and airy apartment; the appointments are also good. Miss Bann, the matron, who was trained at Manchester under Dr. Reid, has held her present appointment for six years. She possesses tact, experience, administrative power, kindliness, and sound judgment, and is undoubtedly an officer whose co-operation any hospital committee would be fortunate to secure. There is a private nursing staff attached to the hospital, which supplies the public with nurses. They have evidently been selected with great care, and exhibit a brightness and intelligence which must make their services especially grateful to the patients. Taking the reconstructed wards as a whole, they may be regarded as well found, efficient, and satisfactory. There is, however, one blot which greatly surprised us, because it is in such striking contrast to every other portion of the institution. We allude to the children's ward, which, from some extraordinary oversight, is allowed to contain fourteen cots instead of ten, although the latter is the maximum number that ought to be allowed if due regard is paid to the responsibilities of those who are at the head of the management of the institution. Formerly, we believe, this ward was much larger, but a good portion has been taken off to form a corridor to connect the main building with the recent extensions. This circumstance may account for the present crowded condition of the children's ward, but we feel constrained to say that no consideration whatever ought to permit the placing of more than ten cots in a space so limited as that now devoted to the children's department.

### Bristol Hospital for Sick Children and Women.

This hospital was established in 1866, thanks to the devoted liberality of its present treasurer, Mr. Mark Whitwill, who displays a whole-hearted devotion to the interests of this charity. No expense has been spared to make the hospital efficient and attractive. The wards are admirably proportioned, the cots and other appliances are excellent, and the whole place strikes the visitor with pleasure, because there is everywhere evidence of attention to every detail calculated to promote the comfort and to expedite the recovery of the sick. The corridors are heated on the Æolus system, which has proved satisfactory after a prolonged trial. In connection with the hospital are detached buildings for the isolation of infectious cases. The children show attachment to their nurses, and their bright and happy faces prove the devoted care which they experience from everybody connected with the management. The Sister in charge of the wards, whose name we do not know, is undoubtedly the right woman in the right place. She has everything well it hand, and it was a pleasure to see on all hands evidence of the success of her system and management.

### General Observations.

Taking these three hospitals together, the visitor must be struck with the admirable spirit which dominates the whole of the resident staff. Everybody seemed bright, happy, intelligent, and active. Few inspections of similar institutions have, in consequence, been more pleasing than those we made at Bristol. Certainly anyone who seeks admission to these institutions when ill will find them pleasant resting places, because here the care of each individual patient is made the predominant feature of the management. From the fact that both the Royal Infirmary and the General Hospital have private nursing staffs in connection with them, the governors would be well advised to communicate with the authorities of the National Pension Fund for Nurses, with a view to securing affiliation on the plan adopted by the London, Winchester, and other hospitals similarly situated. Any hospital which has a private nursing staff must necessarily make a profit out of its nurses, and it is only just and reasonable that a proportion of that profit should be devoted to the purpose of helping the nurses to help themselves to secure an adequate provision against the days of sickness, incapacity, and old age. Having visited Bristol with the feeling that a stranger must necessarily find much to criticise and something to regret, it is our duty to state that this impression was founded on a misapprehension. It arose from a knowledge of the state of affairs which existed years ago, but which has now been remedied, thanks to the intelligence and enterprise of the medical and resident staff, and to the generosity and wise liberality of the committees of management. We understand that both the infirmary and the hospital would like to have a considerable addition to their present income, and we believe that if the Bristol citizens take the trouble to visit their hospitals, and to see for themselves how excellent is the work which they are doing; they will feel moved to promptly supply any funds which may be necessary to accomplish all the good works which the committee and medical staff have in hand.

### The Bristol Eye Hospital.

This hospital, which was established in 1810, has been recently considerably enlarged and improved, owing mainly to the devoted labours of Mr. F. Richardson Cross, the eminent oculist, who is also the surgeon, to this institution. It contains excellent accommodation for the out-patient work; the special system for supplying the patients with spectacles and other appliances being especially good. Further, the cleverness with which the old houses, which now form the hospital buildings, have been converted to their present purpose deserves special notice. With an income of less than £1,000 per annum, this institution relieves some 200 in, and 5,000 out patients every year. It is deservedly popular with the patients, and should secure from the public all the support which its managers may ask for from time to time.

